# Host Responses to Intestinal Microbial Antigens in Gluten-Sensitive Mice

**DOI:** 10.1371/journal.pone.0006472

**Published:** 2009-07-31

**Authors:** Jane M. Natividad, Xianxi Huang, Emma Slack, Jennifer Jury, Yolanda Sanz, Chella David, Emmanuel Denou, Pinchang Yang, Joseph Murray, Kathy D. McCoy, Elena F. Verdú

**Affiliations:** 1 Farncombe Family Digestive Health Research Institute, McMaster University, Hamilton, Canada; 2 Institute of Agrochemistry and Food Technology (IATA), Spanish National Research Council (CSIC), Valencia, Spain; 3 Department of Immunology, Mayo Clinic, Rochester, Minnesota, United States of America; 4 Department of Pathology, McMaster University, Hamilton, Canada; 5 Division of Gastroenterology, Mayo Clinic, Rochester, Minnesota, United States of America; Sun Yat-Sen University, China

## Abstract

**Background and Aims:**

Excessive uptake of commensal bacterial antigens through a permeable intestinal barrier may influence host responses to specific antigen in a genetically predisposed host. The aim of this study was to investigate whether intestinal barrier dysfunction induced by indomethacin treatment affects the host response to intestinal microbiota in gluten-sensitized HLA-DQ8/HCD4 mice.

**Methodology/Principal Findings:**

HLA-DQ8/HCD4 mice were sensitized with gluten, and gavaged with indomethacin plus gluten. Intestinal permeability was assessed by Ussing chamber; epithelial cell (EC) ultra-structure by electron microscopy; RNA expression of genes coding for junctional proteins by Q-real-time PCR; immune response by *in-vitro* antigen-specific T-cell proliferation and cytokine analysis by cytometric bead array; intestinal microbiota by fluorescence in situ hybridization and analysis of systemic antibodies against intestinal microbiota by surface staining of live bacteria with serum followed by FACS analysis. Indomethacin led to a more pronounced increase in intestinal permeability in gluten-sensitized mice. These changes were accompanied by severe EC damage, decreased E-cadherin RNA level, elevated IFN-γ in splenocyte culture supernatant, and production of significant IgM antibody against intestinal microbiota.

**Conclusion:**

Indomethacin potentiates barrier dysfunction and EC injury induced by gluten, affects systemic IFN-γ production and the host response to intestinal microbiota antigens in HLA-DQ8/HCD4 mice. The results suggest that environmental factors that alter the intestinal barrier may predispose individuals to an increased susceptibility to gluten through a bystander immune activation to intestinal microbiota.

## Introduction

Celiac disease (CD) is an immune-mediated enteropathy triggered by the ingestion of gluten containing cereals, and in particular gliadin, the storage protein in wheat. It has recently been recognized that both the pathology and the clinical spectrum of CD varies considerably from severe to subtle, and that the clinical expression is not restricted to the presence of mucosal atrophy [1], [2]. The concept of gluten sensitivity (GS) incorporates a variety of pathologic, immunological, and clinical scenarios that may, or may not, form part of the “celiac” spectrum such as gluten-sensitive diarrhea, immunological mucosal response to gluten in family members of celiac disease, persistent positive specific serology for celiac disease in the absence of defined enteropathy, and subtle immunopathological changes in the intestine exposed to gluten. Typically, these disorders occur in individuals who carry the same HLA genotypes associated with celiac disease-DQ2 and DQ8 [3]–[7]. This has led to the development of animal models of gluten-sensitivity that mimic certain aspects of gluten-induced pathogenesis [8]. HLA-DQ8/HCD4 or single HLA-DQ8 transgenic mice that are sensitized with gluten develop an immune response to gliadin that involves both the adaptive and innate immune system [8]–[11]. Although these gluten-sensitive mice do not spontaneously develop intestinal atrophy, they exhibit gluten-dependent changes in gut neuromuscular and epithelial secretory function [11]. This model has proven useful for the preclinical testing of novel experimental therapies designed to block gluten-induced mucosal pathology [12].

The presence of HLA-DQ2/DQ8 genes are necessary but not sufficient for the development of CD [13], as up to 25–40% of general populations in United States carry these genes and eat gluten, but do not develop a celiac lesion [2], [13], thus raising the possibility of contributing environmental and genetic risk factors yet to be identified [14]. The net availability of gliadin to the lamina propria seems to be an important factor in the inflammatory response of celiac patients. The immobilization and haptenation of gluten components to the extracellular matrix proteins by tissue transglutaminase aids and allows reservoirs of antigenically potentiated gluten components to reach increased concentrations *in vivo*, and may even induce a widespread mucosal response against auto-antigens [15]. Indeed, celiac patients have been shown to increase systemic titres of IgA antibodies against collagen [15].

Under normal conditions, the intestinal epithelium acts as a protective barrier restricting transport of luminal antigens, and only allows small and selective quantities to permeate the mucosa [16]–[18]. In contrast, increased intestinal permeability has been demonstrated in patients with active CD [19], [20] and their healthy relatives, suggesting that in a proportion of cases, intestinal barrier abnormalities may predate overt inflammation [21]. Altered barrier function could be a critical step in facilitating the host responses that contribute to the clinical expression of gluten sensitivity. Thus, the present study was designed to investigate whether alteration of intestinal barrier function using the non-steroidal anti-inflammatory drug (NSAID), indomethacin, enhances gluten-induced epithelial injury and influences subsequent host responses to gut luminal antigens. Our results show that indomethacin enhances gluten-induced changes in the mucosa leading to increased IFN-γ release by gliadin-stimulated splenocytes and to systemic priming against intestinal microbiota antigens. In genetically predisposed hosts with long standing barrier abnormalities, this mechanism may lower the threshold of inflammatory responses to specific antigens.

## Results

### Gluten sensitization and indomethacin treatment led to retardation of weight gain

Gluten sensitized mice and non-sensitized mice treated with indomethacin exhibited a mild retardation of weight gain after 7 weeks compared to non-sensitized controls. Gluten-sensitized mice treated with indomethacin exhibited a more severe retardation of weight gain after 7 weeks, compared to all groups (Figure S1). These results suggest delayed thriving in mice treated with both gluten and indomethacin.

### Indomethacin increased tissue conductance and permeation of macromolecules across epithelium in gluten-sensitized mice

**Figure 1:**
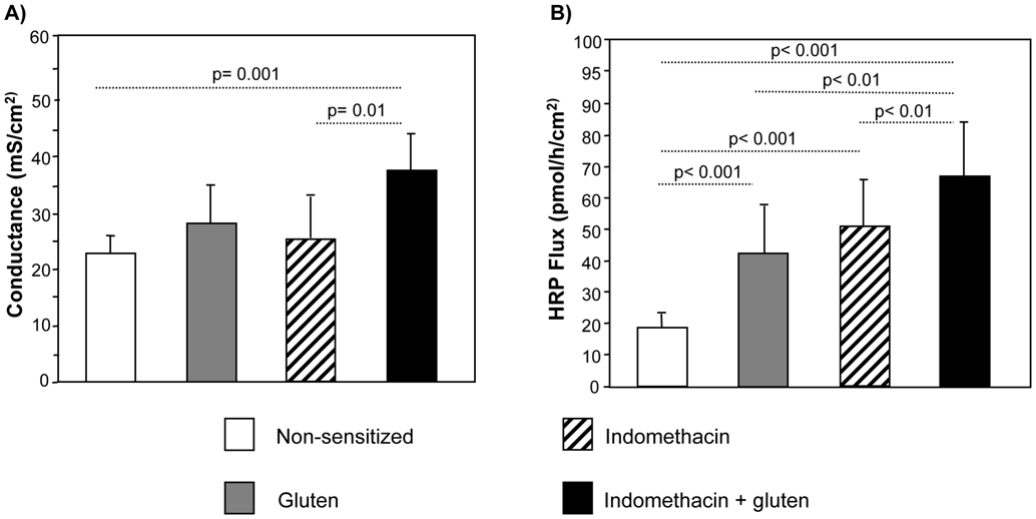
Intestinal barrier measurements. Ussing-chamber experiments were performed on jejunum from all four groups 24 hours after the last gluten challenge. (A) Gluten-sensitized mice treated with indomethacin showed a significant increase in tissue conductance. (B) HRP flux (transcellular permeability) increased significantly in all treatment groups compared to non-sensitized controls, however the highest values were observed in gluten plus indomethacin treated mice. Data represent the means±SEM of 10 mice/group.

In order to determine the effects of gluten sensitization and indomethacin treatment on intestinal permeability, tissue conductance and HRP flux were measured in segments of small intestine. Gluten-sensitized mice treated with indomethacin exhibited a significant increase in small intestinal tissue conductance compared to non-sensitized controls and indomethacin alone-treated mice (Figure 1A). HRP flux, a measurement of transcellular macromolecular transport, was elevated in all groups compared to non-sensitized controls. Gluten sensitization and indomethacin treatment, however, led to the highest increase in HRP flux with approximately 2.5 fold increase compared non-sensitized controls (Figure 1B). The potentiation of intestinal permeability changes by indomethacin was not observed in C57BL/6 mice sensitized with gluten, stressing the importance of the DQ8 transgene in the model (Figure S2).

### Indomethacin led to epithelial ultra-structural damage in gluten-sensitized mice

**Figure 2:**
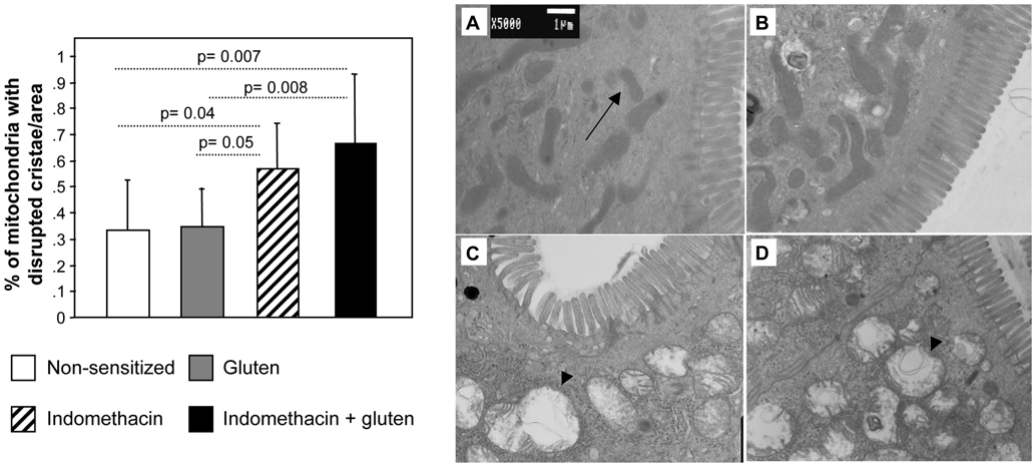
Evaluation of mitochondrial disruption. Mitochondrial ultra-structure was assessed by electron microscopy. Indomethacin increased the fraction of disrupted mitochondria. Gluten sensitization plus indomethacin treatment further increased the proportion of altered mitochondria. Data represent the means±SEM of 6 mice/group. Reperesentative pictures from (A) Control mouse, arrow: normal mitochondria; (B) Gluten-sensitized mouse; (C) Indomethacin-treated mouse, arrowhead: mitochondria with disrupted cristae; (D) Indomethacin-treated plus gluten-sensitized mouse, arrowhead: mitochondria with disrupted cristae.

**Figure 3:**
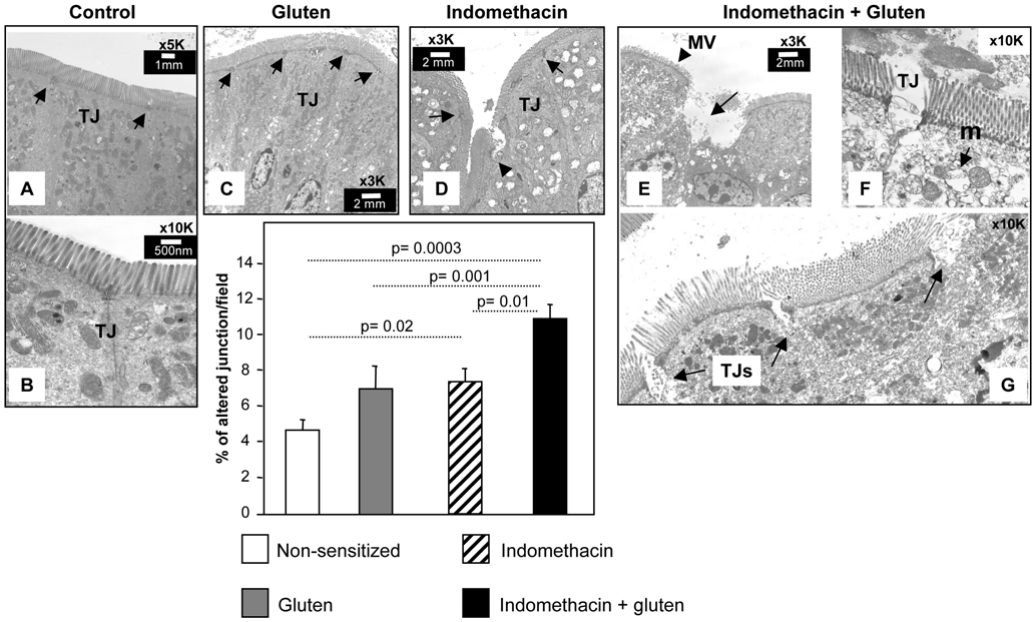
Apical epithelial cell structural abnormalities. Epithelial ultra-structure was assessed by electron microscopy. A significant proportion of altered TJ was observed in gluten-sensitized plus indomethacin-treated mice. Indomethacin alone also increased the proportion of altered TJ but to a lesser extent than indomethacin plus gluten. Gluten sensitization alone tended to increase the proportion of altered TJ but this did not achieve statistical significance (p = 0.09 vs non-sensitized controls). Data represent the means±SEM of 5 mice/group. Representative pictures of (A–B) a control mouse, arrow: tight junction (TJ) with preserved structure; (C) Gluten-sensitized mouse, arrow: TJ with preserved structure; (D) Indomethacin-treated mouse showing one altered TJ (arrowhead) and 2 junctions with normal struture (arrows); (E–G) Gluten plus indometacin treated mouse; (E) arrowhead: microvilli (mv) height reduction, arrow: apical epithelial cell destruction; (F) Altered TJ, arrow: mitochondria (m) with disrupted cristae; (G) Several altered TJs.

Intestinal morphology was analyzed using electron microscopy. No mitochondrial abnormalities were detected in non-sensitized controls or in gluten-sensitized mice without indomethacin (Figure 2A,B). Mitochondrial abnormalities were observed in mice treated with indomethacin alone (Figure 2C) and in gluten-sensitized mice treated with indomethacin (Figure 2D). We quantified the proportion of mitochondria with disrupted cristae in a defined area with approximately the same number of mitochondria (Figure 2E). Gluten-sensitized mice treated with indomethacin, had a higher proportion of damaged mitochondria than gluten-sensitized mice without indomethacin. Epithelial cell edema and disrupted microvilli were observed in tissues obtained from gluten-sensitized plus indomethacin treated mice but not from the rest of the groups (Figure 3). These marked ultra-structural changes were not observed in gluten-sensitized (Figure 3C) and indomethacin alone-treated mice (Figure 3D). Altered junctional ultra-structure was more pronounced in tissues from gluten-sensitized plus indomethacin-treated mice (Figure 3E–G) compared to non-sensitized (A–B), gluten-sensitized (C) and indomethacin alone-treated mice (D).

### Concomitant treatment with indomethacin and gluten led to reduction of E-cadherin mRNA expression

**Figure 4:**
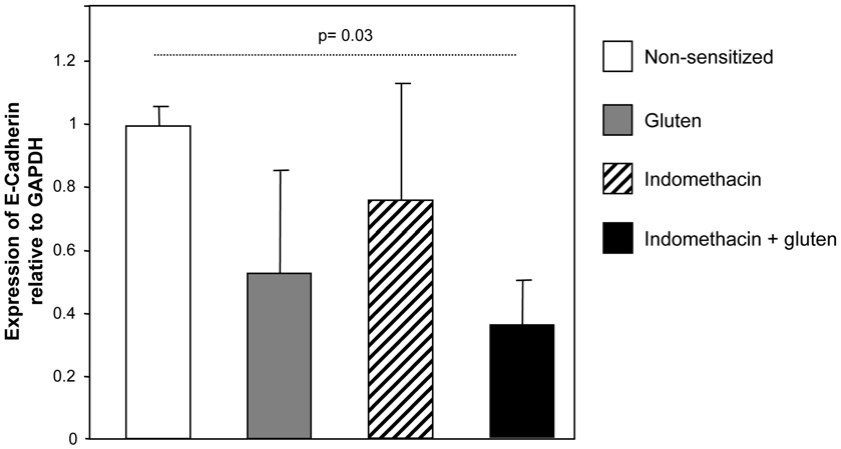
RNA level of E-cadherin relative to control (non-sensitized). Real-time QPCR experiments were performed on jejunum collected from all groups 24 hours after the last gluten challenge. Gluten-sensitized and indomethacin alone-treated mice showed a trend for decreased expression of E-cadherin relative to non-sensitized controls. Gluten-sensitized plus indomethacin-treated mice showed marked down-regulation of E-cadherin RNA level relative to non-sensitized controls. Data represent the means±SEM of 6 mice/group.

The changes in the ultra-structure of the tight junctions prompted us to investigate whether there were alterations in RNA expression of epithelial adherens and tight junctional proteins. Gluten-sensitized and indomethacin alone-treated mice showed reduced E-cadherin RNA expression by a mean factor of 0.762 and 0.533 respectively, but this did not achieve statistical difference relative to non-sensitized controls (Figure 4). In contrast, expression of E-cadherin RNA was markedly down regulated by 2.75 fold compared to non-sensitized mice, in gluten-sensitized plus indomethacin-treated mice. Gluten sensitization and indomethacin did not affect significantly the relative RNA expression of tight junction ZO-1 (Figure S3).

### Indomethacin treatment affected the release of IFN-γ by splenocytes from gluten-sensitized mice after *in vitro* challenge with PT-gliadin

**Figure 5:**
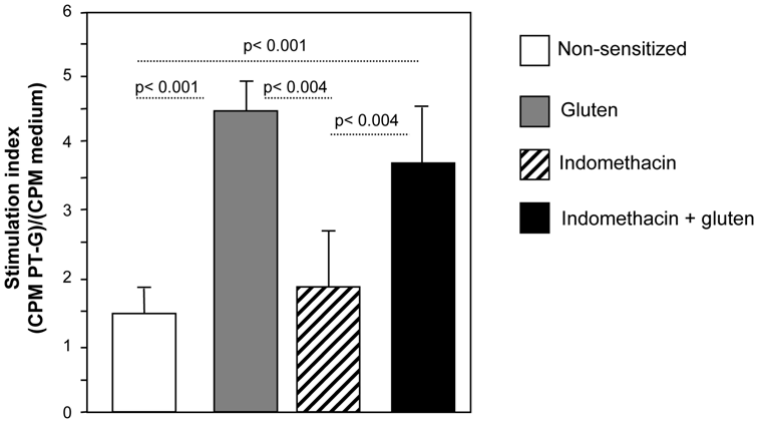
Splenocyte proliferation after incubation with PT gliadin. Proliferation was measured by ^3^H-thymidine incorporation and expressed as stimulation index. Splenocytes from gluten-sensitized mice treated with or without indomethacin exhibited increased proliferation compared to non-sensitized controls. Data represent the means±SEM of 6 mice/group.

In order to assess whether the increase in permeability and the damage to the intestinal structure in gluten-senstized mice after treatment with indomethacin led to an increase in the systemic immune response to gliadin, we analyzed antigen-specific proliferation and cytokine production of splenocytes. Increased T cell proliferation after incubation with PT-gliadin was observed in gluten-sensitized mice, but not in non-sensitized controls (Figure 5). Differences in proliferation did not reflect cell death or an inability to proliferate as polyclonal stimulation with ConA led to equal responses in all groups (data not shown). Surprisingly, indomethacin-treatment of gluten-sensitized mice did not exhibit higher levels of antigen-specific proliferation compared to gluten-sensitized mice that were not given indomethacin (Figure 5). *In-vitro* incubation of splenocytes from gluten-sensitized mice with indomethacin did not increase cell proliferation (Figure S4).

**Figure 6:**
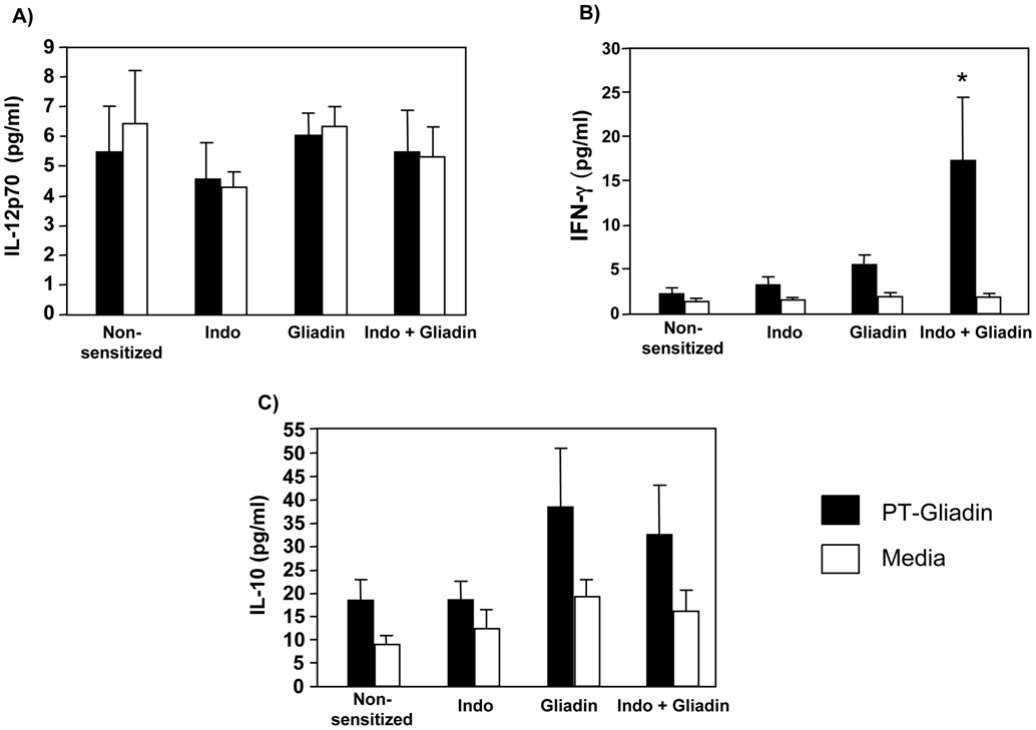
Cytokines in supernatant of splenocyte cultures after incubation with PT-gliadin (black) or medium (white). Expressions of (A) IL-12p70, (B) IFN-γ, (C) IL-10 were determined by CBA analysis. Culture supernatants from gluten-sensitized plus indomethacin (Indo) treated mice showed increased IFN- γ (*p<0.01 vs all groups). Cultured splenocytes from gluten-sensitized mice, with or without indomethacin showed a trend for increased IL-10 release after PT-gliadin stimulation (p = 0.09). Data represent the means±SEM of 6 mice/group.

To further assess the systemic immune response IL-12, IFN-γ and IL-10 levels in the supernatant of the PT-gliadin stimulated splenocytes cultures were determined (Figure 6). Whilst IL-12 was not induced above media alone, IL-10 levels were slightly increased in the culture supernatant of splenocytes from gluten-sensitized and indomethacin-treated gluten-sensitized splenocytes after *in vitro* stimulation with PT-gliadin, although the increases were not statistically significant.

In contrast, indomethacin treatment of gluten-sensitized mice led to a significant increase in IFN-γ production in response to PT-gliadin stimulation. *In-vitro* incubation of splenocytes from gluten-sensitized mice with indomethacin did not increase IFN-γ production (Figure S5).

### Gluten and indomethacin led to changes in intestinal microbiota composition

**Figure 7:**
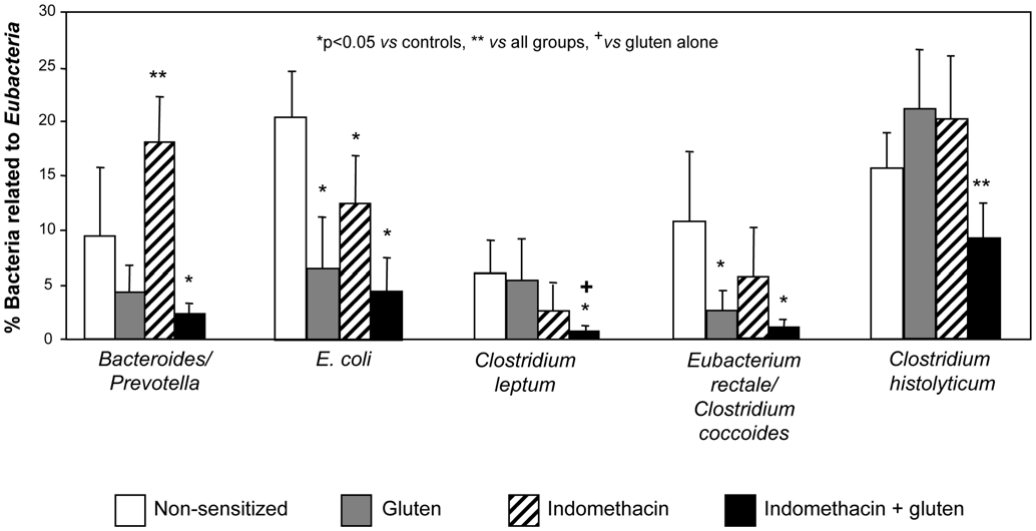
Microbiota composition. Using 9 different oligonucleotide probes and fluorescent *in situ* hybridization (FISH), microbial profile was investigated in the distal jejunum of sensitized mice with and without indomethacin. The results indicate a significant perturbation in the proportions of microbiota investigated in all 3 treatment-groups when compared to non-sensitized controls, and remarkably in the gluten-sensitized plus indomethacin group. These differences achieve statistical significance in Bifidobacteria (*p = 0.04 vs controls, +p = 0.03 vs gluten) and Clostridium Leptum cluster (both *p = 0.02 vs controls and gluten sensitized, **p = 0.04 vs indomethacin) compared to gluten-sensitized alone. Data represent the means±SEM of 6 mice/group.

We next analyzed whether gluten sensitization or indomethacin treatment could lead to changes in the composition of the intestinal microflora. Gluten-sensitized mice showed a significant decrease of gut bacterial proportions of *E. coli* and *E. rectale-Clostridium* groups, as compared to control mice. Indomethacin-treated mice also showed reductions in *E. coli* proportions, but increases in those of *Bacteroides-Prevotella* group. Gluten-sensitized mice treated with indomethacin showed the most remarkable alterations in the intestinal microbiota, characterized by reductions in the relative abundance of all bacterial groups analysed as compared with control mice (Figure 7).

### Indomethacin led to systemic priming against intestinal microbiota in gluten-sensitized mice

**Figure 8:**
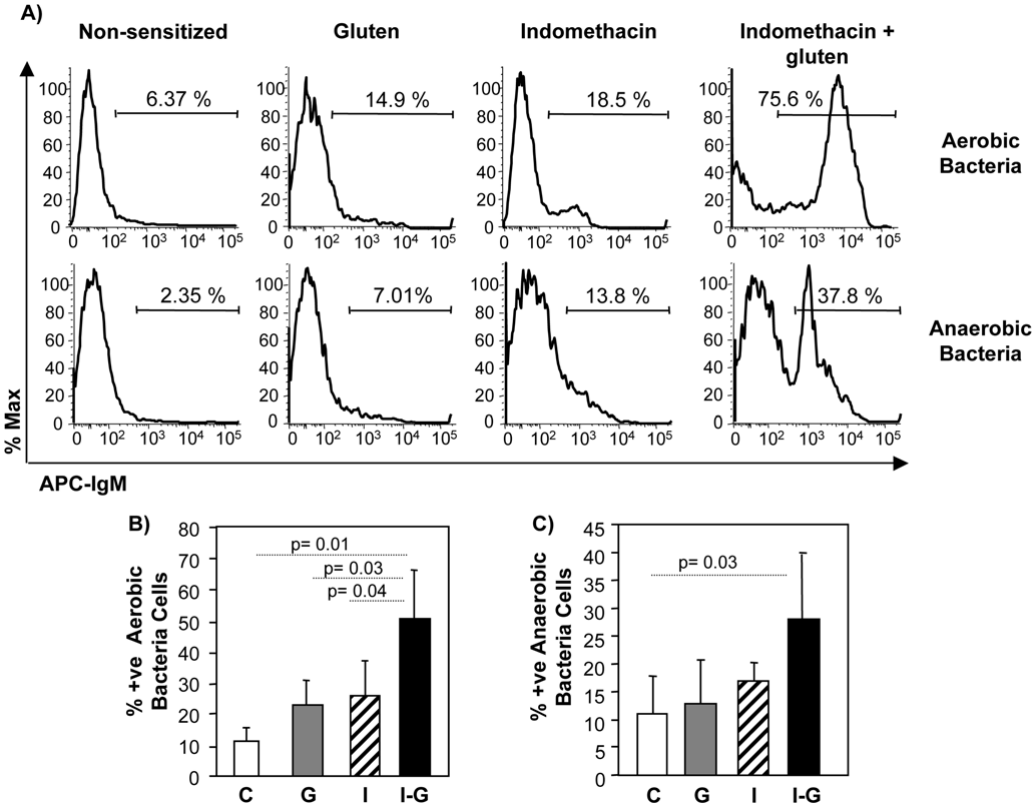
Systemic antibodies against commensals. Serum from indomethacin and gluten treated mice showed significant positive serum antibodies against their aerobic and anaerobic intestinal microbiota. (A) Representative FACS histogram from each treatment gated on IgM^+^ cells; (B) Proportion of IgM^+^ aerobic bacterial cells for each treatment groups; (C) Proportion of IgM^+^ anaerobic bacterial cells for each treatment groups. Data represent the means±SEM of 6 mice/group.

Previous data suggests that bacterial translocation beyond the mucosal immune system is necessary for systemic priming to intestinal commensals [26]. To determine if the increased conductance and HRP flux induced by gluten-sensitization and indomethacin was accompanied by loss of the host's normal systemic ignorance to the intestinal microbiota, we measured specific IgM antibody responses to culturable aerobic or anaerobic commensals. Non-sensitized mice showed no evidence of IgM specific antibodies against aerobic and anaerobic commensal flora as assessed by flow cytometric analysis of anti-bacterial IgM responses (Figure 8 & Figure S6). Treatment with either gluten or indomethacin alone led to the production of very low titres of IgM antibodies directed against a subset of culturable bacteria. Gluten sensitization plus indomethacin treatment, however, resulted in induction of strong specific IgM responses directed against 40–80% of culturable bacteria. The anti-bacterial IgM induced was specific to the commensal microflora of the host and did not bind to Salmonella, which these mice have never been exposed to (Figure S7). These data indicate that the combination of gluten-sensitization and increased intestinal permeability as induced by indomethacin treatment increased systemic priming to the commensal microflora.

## Discussion

The aim of this study was to investigate whether modulation of the intestinal barrier by an environmental trigger can affect host responses to luminal antigens in a genetically predisposed host. Our results demonstrate that gluten sensitization and long-term gluten challenge in HLA-DQ8/HCD4 mice alters intestinal permeability as assessed by increased transcellular macromolecular transport and a tendency for higher conductance values (paracellular pathway). This is in agreement with previous reports showing that gluten peptides have the ability to rapidly disrupt the apical junctional structure [22]–[25], and can also be transported by an abnormal transcellular route [26]. Administration of indomethacin to gluten-sensitized mice led to more pronounced barrier dysfunction, which was accompanied by a mild pro-inflammatory shift with marked changes in EC ultra-structure, reduced E-cadherin mRNA levels in the proximal small intestine and generation of systemic antibody responses to intestinal microbiota.

Indomethacin has been used at higher doses as a model of inflammatory bowel disease (IBD) [27], [28], and has been shown to compromise epithelial barrier integrity and function, induce motility changes, and promote small intestinal bacterial overgrowth and translocation [27], [29]. In the present manuscript, low-dose indomethacin induced permeability changes without producing macroscopic or microscopic ulcers. However, ultra-structural observations revealed epithelial cell abnormalities characterized by mitochondria with disrupted cristae in mice receiving indomethacin. Mucosal toxicity induced by gluten in HCD4/DQ8 mice was potentiated by indomethacin, as shown by more marked elevation of HRP flux and a significant increase in tissue conductance. After administration of indomethacin, C57BL/6 mice exhibited increased HRP flux, but no change in tissue conductance. Gluten sensitization however, did not induce barrier dysfunction in C57Bl6 mice, emphasizing the relevance of the DQ8 transgene in the model (Figure S2). Electron microscopy examination in gluten-sensitized HCD4/DQ8 mice treated with indomethacin revealed more structural abnormalities in the apical region of the epithelium compared to gluten alone-treated mice. Furthermore, RT-PCR analysis demonstrated reduced E-cadherin RNA levels in gluten-sensitized plus indomethacin treated mice. E-cadherin is required for TJ formation and there is growing evidence for its role as modulator of TJ and intestinal barrier function [30], [31]. E-cadherin expression is reduced in children with CD and gliadin has been shown to alter its expression [32]. Our results support the hypothesis that both gluten and indomethacin play a role in the expression of E-cadherin, and that this effect is potentiated in a genetically susceptible host when both agents are administered together. Marked reduction of E-cadherin may constitute a mechanism for the enhanced barrier dysfunction observed in gluten-sensitized and indomethacin-treated HCD4/DQ8 mice.

The marked changes in barrier function in gluten-sensitized plus indomethacin-treated mice were accompanied by increased IFN-γ production in splenocyte cultures after incubation with PT-gliadin. These results suggest a shift towards a mild systemic pro-inflammatory state. Previous studies have shown that cyclooxygenase-2 (COX-2)-dependent arachidonic acid metabolites are important in the maintenance of intestinal immune homeostasis, particularly in the immunoregulation of dietary antigens [33]. Consequently, COX-2 inhibitors such as indomethacin may exacerbate the immune response to dietary antigens [33]. Our results using *in vitro* incubation of splenocytes with PT gliadin and indomethacin, however, do not support a direct effect of indomethacin on splenocyte proliferation and IFN-γ release. Thus, we hypothesize that the shift in the immune response may be due to an enhanced uptake of luminal contents, including commensal bacteria, through a more structurally damaged and permeable epithelium.

The intestinal epithelium regulates permeation of luminal antigens and excessive immune activation within the mucosa [16], [34]. The marked barrier defect in gluten-sensitized mice treated with indomethacin may not only allow an increased influx of gliadin peptides across the epithelium but also of other luminal antigens such as intestinal microbiota with potential bystander, pro-inflammatory effects. Germ-free rats have been reported to have a higher threshold for intestinal damage after indomethacin administration compared to specific pathogen-free (SPF) rats [35]. Since inhibition of prostaglandins in the absence of an intestinal microbiota is less severe, the results raise the hypothesis that intestinal bacteria potentiate the development of indomethacin-induced mucosal lesions. Thus, dysmotility induced by gluten sensitization [11] or indomethacin [36], and/or the ability of indomethacin to induce small intestinal dysbiosis [36], [37] may facilitate bacterial translocation. Due to a severely impaired intestinal barrier in both gluten-sensitized and indomethacin-treated mice, increased permeation of luminal bacteria may disturb the natural commensal homeostasis in the gut promoting a pro-inflammatory response. SPF mice have been shown to be systemically ignorant to their intestinal microbiota due to the geographic and functional separation between the mucosal and systemic immune system by the mesenteric lymph nodes (MLN) [17], [38]. Our results show, however, that a low level of systemic priming against intestinal microbiota occurs in SPF mice treated with either gluten or indomethacin alone. Gluten-sensitized mice, in which barrier function is further perturbed by indomethacin treatment, show dramatic systemic priming to their intestinal microbiota. These data therefore imply that gluten-sensitization, in combination with indomethacin treatment, results in decreased mucosal containment of the commensal flora. NSAIDs have been shown to reduce the phagocytic properties of macrophages [39]. Thus, we acknowledge that it is possible that in addition to changes in intestinal barrier function, indomethacin may have a dual effect by inhibiting macrophage function, allowing the persistence of live bacteria, and facilitating a systemic immune response against intestinal microbiota. F4/80^+^ cell counts in the lamina propria of gluten plus indomethacin-treated mice were significantly increased (Figure S8, Protocol S1), however macrophage function was not assessed. The exact identities of the commensals to which gluten and indomethacin-treated mice are systemically primed in this model are not yet known but the absence of IgM binding to *Salmonella*, known to be absent from the commensal flora of our mice, strongly suggests the specificity of the IgM antibodies against commensal flora in our mice (Figure S7). The clinical relevance of the loss of systemic ignorance against the intestinal microbiota remains to be established, however, systemic priming to the commensal flora represents a significant shift in the normal relationship between host and commensal bacteria [40]. Consequently, this may indicate a novel mechanism that could contribute to the progression of disease in a gluten-sensitive host. On the other hand, specific IgM against flora may be part of a protective mechanism mounted by the immune system to limit subsequent translocation and widespread inflammation. Additional host factors, such as an underlying immune dysbalance, may play a role in determining whether this mechanism will become maladaptive and contribute to widespread inflammation. A recent epidemiological study has determined that consumption of non-steroidal anti-inflammatory drugs (NSAIDs) is a risk factor for the development of irritable bowel syndrome [41]. No epidemiological studies to date have investigated whether a history of NSAID consumption is also a risk factor for the protean clinical expression in gluten sensitivity.

Although the role of the intestinal microbiota in other chronic diseases of the gut is clearly established [for review see 42] little is known about the role of abnormal immune responses to commensals in gluten and other food intolerances. Recent findings, however, report presence of rod-shaped bacteria in the mucosa of active and non-active celiac patients but not in healthy controls [43]. A study in patients with CD revealed the presence of serological responses to microbial antigens, such as anti-*Saccharomyces cerevisiae*, anti-I2 (*Pseudomonas fluorescens*) and anti-ompW, compared to healthy controls. Interestingly, microbial seropositivity was also present in gluten-sensitive patient with no evidence of active CD. However, increasing age was associated with sero-reactivity for anaerobic bacteria, possibly reflecting exposure to different environmental antigens with longer duration of disease [44]. The disappearance of anti-*Saccaromyces cervisiae*-antibodies (ASCA) after a gluten-free diet suggests that healing of mucosal lesions is related to microbial sero-markers [45]. However, a causal relationship between gut dysfunction, symptoms and microbial sero-responses in CD remains to be determined. It is possible that accumulated bacterial products have a bystander effect and lower the threshold for immune cell activation [46], [47]. To this respect, a study in DQ8 mice has shown that oral challenge with *Lactobacillus casei* at the time of mucosal sensitization with gliadin and cholera toxin exacerbates the Th1 response induced in the model [48]. Thus, it is possible that dysbiosis or shifts in the composition of the intestinal microbiota at the time of gluten sensitization, and not necessarily the presence of a pathogen, contribute to enhance gluten-induced immune responses. An altered microbiota composition has been reported in patients with CD compared to healthy controls [49], [50]. In this study, we observed significant alterations in the composition of the small intestinal microbiota in gluten-sensitized mice treated with indomethacin. It is unclear, however, if these changes are primary or secondary to the functional gut abnormalities observed in the model [11].

In conclusion, our findings suggest that an environmental alteration of the intestinal barrier plays a critical role in determining host immune responses to gluten and intestinal microbiota antigens. Bystander luminal antigens such as components of the intestinal microbiota may contribute to enhance inflammatory responses to dietary antigens such as gluten. This mechanism may become important in genetically predisposed hosts with longstanding barrier abnormalities. The results warrant further investigations on the interactions between host genotype, diet, and intestinal microbiota.

## Materials and Methods

### Animals

All experiments were conducted with approval from the McMaster University Animal Care Committee. Male transgenic mice expressing HLA-DQ8 genes (HLA-DQA1*0301; HLA-DQb1*0302) in the absence of endogenous mouse class II genes or HLA-DQ8/HCD4 double transgenic mice were used [8], [51]. The mice were bred in a conventional specific pathogen free colony (SPF) at McMaster University and maintained for at least 2 generations prior to breeding on a gluten-free diet (Bio-Serv, New Jersey). Mice were used at the age of 8-14 weeks. Male C57BL/6 mice were purchased from Taconic (Hudson, NY, USA) (supplementary data).

### Sensitization protocol and indomethacin treatment

All mice were continuously fed with a gluten-free diet and water available *ad libitum*.

Mice were sensitized by injecting intraperitoneally (ip) 500 µg of gluten (Sigma-Aldrich, Ontario) dissolved in 0.02 mM acetic acid in 50 µl of Complete Freund's Adjuvant (CFA, Sigma-Aldrich, Ontario). One week after sensitization, gluten challenge was performed 3 times on a weekly basis by intragastric gavage, for 7 weeks, using 2 mg of gluten dissolved in 0.02 mM acetic acid. Indomethacin was administered by gavage (Ovation Pharmaceuticals, Ontario) (3.5 mg/kg) 24 hours before the gluten challenge. Control groups consisted of a) non-sensitized mice (CFA only) subsequently gavaged with rice cereal (2 mg/0.02 mM acetic acid), b) gluten-sensitized mice subsequently gavaged with gluten (2 mg/0.02 mM acetic acid) c) non-sensitized mice (CFA only) subsequently gavaged with indomethacin (3.5 mg/kg).

### In vitro intestinal permeability

Two sections of jejunum from each mouse were used for Ussing chamber studies. Briefly, 5 cm of jejunum samples were collected and divided into 2 segments. Each segment was opened along the mesenteric border, flattened and mounted in an Ussing chamber with an opening of 0.6 cm^2^. Tissues were bathed in oxygenated Krebs buffer containing 10 mM glucose (serosal side) or 10 mM mannitol (luminal side) at 37°C. After a 20-minute equilibration period, conductance (G: mS/cm2) were recorded. Mucosal to serosal transport of macromolecules was assessed by adding horseradish peroxidase (HRP; type II, Sigma-Aldrich, Ontario), a commonly used macromolecular marker, in the luminal side. Serosal samples (500 µl) were obtained at 30 minutes intervals for 2 hours. Intact HRP was assessed using a modified Worthington method with *o-*dianosidine dihydrochloride (Sigma-Aldrich, Ontario) as the substrate, and mucosal to serosal fluxes were calculated according to standard formulae and expressed as pmol/cm^2^/hr.

### Examination of epithelial cell injury

Jejunal sections were obtained and immediately fixed in 2.5% glutaraldehyde in 0.1 mol/L sodium cacodylate buffer (pH 7.4) for 2 hours, transferred to sodium cacodylate buffer and stored at 4°C overnight. Tissues were subsequently processed for electron microscopy, and photomicrographs were prepared. Ultra-structural epithelial damage was evaluated in enterocytes by the presence of alterations in brush border, mitochondrial edema and tight junction (TJ) morphology. Epithelial damage was determined by transmission electron microscope (JEOL, Tokyo) of enterocytes in sections from 4–6 animals in each of the 4 study groups. The number of mitochondria with disrupted cristae within the apical region of enterocytes were counted on coded 5000x magnification photomicrographs measuring a total of 300 µm^2^ (125–250 mitochondria/photomicrographs, 5 photomicrographs/mouse, 6 mice/group) using Adobe CS3 Extended (Adobe Systems Incorporated, California). The fraction of altered mitochondria, defined as number of mitochondria with disrupted cristae divided by total number of mitochondria in a view, was calculated. Mitochondria on the edge of micrographs were excluded for evaluation, since neither their boundaries nor area could be accurately determined. The fraction of disrupted TJ structure was calculated as total altered TJ divided by total number of TJ evaluated in 20 fields per mouse in a blinded manner (3–20 tight junctions/field, 60–400 tight junctions/mouse, 4 mice/group). A field is defined as one square in the EM grid, measuring 8100 µm^2^.

### Apical junctional analysis by quantitative real-time PCR

Total RNA from a 30–60 mg proximal small intestine section was isolated using RNeasy mini kit (Qiagen, Ontario). cDNA was synthesized from 2 µg of purified total RNA using M-MLV reverse transcriptase (Invitrogen, Ontario). Quantitative real-time PCR was performed with 1∶20 dilutions of cDNA. The reaction consisted of iQ™ SYBR Green Supermix (Bio-rad, Ontario) for quantitative PCR, primers at 0.5 µM, and 1 µl of cDNA. Amplification was performed using iQ5 Real-Time Detection System at 95°C for 3 min followed by 37 cycles at 94°C for 15 s, 55°C (ZO-1) or 58°C (E-Cadherin) or 59°C (GAPDH) for 20 s, and 72°C for 25 s. Q Real-time PCR was performed using the following primers: ZO-1 5′-AGGACACCAAAGCATGTGTGAG-3′/and 3′-GGCATTCCTGCTGGTTACA-5′;

E-Cadherin 5′-GCACATATGTAGCTCTCATC-3′ and 5′CCTTCACAGTCACACACATG-3′. GAPDH 5′-CCATGGAGAAGGCTGGGG-3′ and 5′-CAAAGTTGTCATGGATGACC-3′ was used as housekeeping gene. CT values reported by iQ5 software were used in the study. PCR efficiencies for each amplicon were determined by making 10-fold serial dilutions of cDNA and then amplifying the cDNA using primers to both the gene of interest and housekeeping gene. Relative expression levels were calculated using the Pfaffl method [52], with efficiency correction for each primer set, using REST software [52]. A melting curve analysis was performed by heating the reactions from 50° to 99°C in 0.2°C intervals while monitoring fluorescence.

### Splenocyte proliferation, cell cultures and cytokine analysis

Peptic-tryptic digests of gliadin (PT-gliadin) was prepared as described previously [54]. Spleen cells were harvested and cultured (4×10^5^ cells/well) in 96-well tissue culture plates at 37°C, 5% CO_2_ for 72 h in the presence or absence of 500 µg/ml PT-gliadin and/or 5 µg/ml indomethacin. The cultures were pulsed with 1 µCi/well [^3^H]-thymidine for 18 h. The cultured cells were harvested onto glass fibre filters using Filtermate harvester (Cambridge Technology, Massachusetts). The radioactivity incorporated was determined with a Beta Scintillation Counter (Beckman, California). Results are expressed as stimulation index (SI) and calculated as: SI = (mean cpm of triplicate cultures containing antigen)/(mean cpm of cells cultured with medium alone).

Splenocyte supernatants were collected 48 h after incubation with or without PT-gliadin and/or indomethacin. The presence of pro-inflammatory cytokines in the supernatant was measured using pro-inflammatory CBA kit (BD Bioscience, California) and analyzed using BD FACSarray Bioanalyzer System (BD Bioscience, California).

### Fluorescent in situ hybridisation (FISH)

Oligonucleotide probes are summarized in Table S1. The group-specific probes were labeled at the 5′-end with fluorescein isothiocyanate (FITC), showing green fluorescence. EUB 338 probe, targeting conserved sequences within the bacterial domain, was used as positive control [55]. NON EUB 338 probe was used as negative control to eliminate the background fluorescence [56]. Both control probes were labelled at the 5′end with either the indocyanine dye Cy3, showing red fluorescence, or with FITC. Aliquots of 36 µl fixed samples were incubated with 4 µl of each fluorescent probe (50 ng/µl) in hybridization solution (10 mM Tris–HCl, 0.9 M NaCl, pH 8.0, and 10% [w/v] sodium dodecyl sulphate) at appropriate temperature (45–50°C) overnight. Afterwards, bacterial cells were incubated with 400 µl washing solution (10 mM Tris–HCl, 0.9 M NaCl, pH 8.0) at 50 °C for 30 min to remove non-specific binding of the probes. Hybridized cells were finally pelleted by centrifugation (12 000 g for 5 min) and resuspended in 400 ml of PBS for flow cytometry detection. Bacterial groups were enumerated by combining each FITC-labelled group-specific probe with the EUB 338-Cy3 probe, and expressed as a ratio of cells hybridizing with the FITC-labelled specific probe to cells hybridizing with the EUB 338-Cy3 probe. This proportion was corrected by subtracting the background fluorescence obtained with the negative control probe NON EUB 338 [57], [58]. Flow cytometry detections were performed using anEPICS® XL-MCL flow cytometer (Beckman Coulter, Florida, USA) as previously described [58]. This instrument is equipped with two light scatter detectors that measure forward (FSC) and side scatter (SSC) and fluorescence detectors that detect appropriately filtered light at green (FL1, 525 nm) and red-orange (FL3, 620 nm) wavelengths. The event rate was kept at the lowest setting (200–300 events per second) to avoid cell coincidence. A total of 15, 000 events were recorded in a list mode file and analyzed with the System II V.3 software (Beckman Coulter).

### Statistical Analysis

Statistical analysis was performed using ANOVA with post-hoc test for simple and multiple comparisons, respectively. Nonparametric statistical significance of relative RNA expression was calculated with REST software [53] by a pairwise fixed reallocation randomization test with 50,000 repeats. Data were presented as means±standard error (SEM).

## Supporting Information

Figure S1 Weight gain over 7-week period. Both gluten-sensitized and indomethacin treated mice exhibited a decreased rate of weight gain compared to non-sensitized controls. Gluten-sensitized mice treated with indomethacin exhibited more pronounced weight gain retardation compared to controls and to gluten-sensitized and indomethacin treated mice. Data represent the means±SEM of 10 mice/group.
(0.08 MB TIF)

Figure S2 Conductance and HRP flux in C57Bl/6 mice. Gluten and/or indomethacin treatment did not lead to changes in tissue conductance. HRP flux was increased in indomethacin treated mice, but not in gluten sensitized mice without indomethacin. Data represent the means±SEM of 10 mice/group.
(0.09 MB TIF)

Figure S3 ZO-1 RNA expression relative to non-sensitized controls. No significant differences were seen when RNA expression for each treatment group was analyzed relative to non-sensitized controls. Data represent the means±SEM of 6 mice/group.
(3.00 MB TIF)

Figure S4 Splenocyte proliferation after incubation with PT-gliadin and/or indomethacin. Stimulation with indomethacin alone did not increase splenocyte proliferation in gluten-sensitized mice. In-vitro stimulation with both PT-gliadin and indomethacin, did not further enhance cell proliferation compared to PT-gliadin alone. Data represent the means±SEM of 6 mice/group.
(0.07 MB TIF)

Figure S5 IFN-γ levels in supernatant of cultured splenocytes after incubation with PT-gliadin and/or indomethacin. Stimulation with indomethacin alone did not increase IFN-γ production in gluten-sensitized mice. In-vitro stimulation with PT-gliadin and indomethacin did not increase IFN-γ levels compared to PT gliadin alone. Data represent the means±SEM of 6 mice/group. ND = not detectable.
(0.07 MB TIF)

Figure S6 Systemic antibodies against commensals. Gluten-sensitized plus indomethacin-treated mice exhibited increased serum antibodies against aerobic and anaerobic bacteria as assessed by median fluorescent intensity signal of APC-labelled anti-IgM (1∶20 serum dilution). Negative controls include serum (−): no serum and bacteria (−): no bacteria. Data represent the means±SEM of 6 mice/group.
(0.11 MB TIF)

Figure S7 Positive and negative systemic antibodies against commensals. (A) Salmonella M557, which is a pathogen not present in our HLA-DQ8/HCD4 mice colony, was stained with serum antibodies from indomethacin plus gluten treated mice. Results show the absence of positive antibodies against Salmonella, thus the specificity of the technique. (B) Salmonella M557 was stained with serum antibodies from Salmonella M557 infected mice. Results show the absence of positive antibodies against Salmonella.
(0.09 MB TIF)

Figure S8 Immunohistochemistry for F4/80+ cells. Staining for F4/80+ was increased in gluten sensitized mice. Infiltration of F4/80+ cells was most marked in gluten-sensitized mice treated with indomethacin. Data represent the means±SEM of 6 mice/group. Representative picture of macrophage infiltration in the lamina propria from (A) control mice (B) gluten-sensitized mice (C) indomethacin-treated mice (D) gluten-sensitized plus indomethacin treated mice.
(0.47 MB TIF)

Table S1 Oligonucleotide probes and hybridization conditions used in FCM-FISH analysis of intestinal bacteria.
(0.06 MB DOC)

Protocol S1 Immunohistochemistry for macrophages.
(0.03 MB DOC)
