# Supplementary figures and images for: Host Responses to Intestinal Microbial Antigens in Gluten-Sensitive Mice
Source: PLoS One. 2009 Jul 31;4(7):e6472. doi: 10.1371/journal.pone.0006472 (PMC2715133; doi:10.1371/journal.pone.0006472)

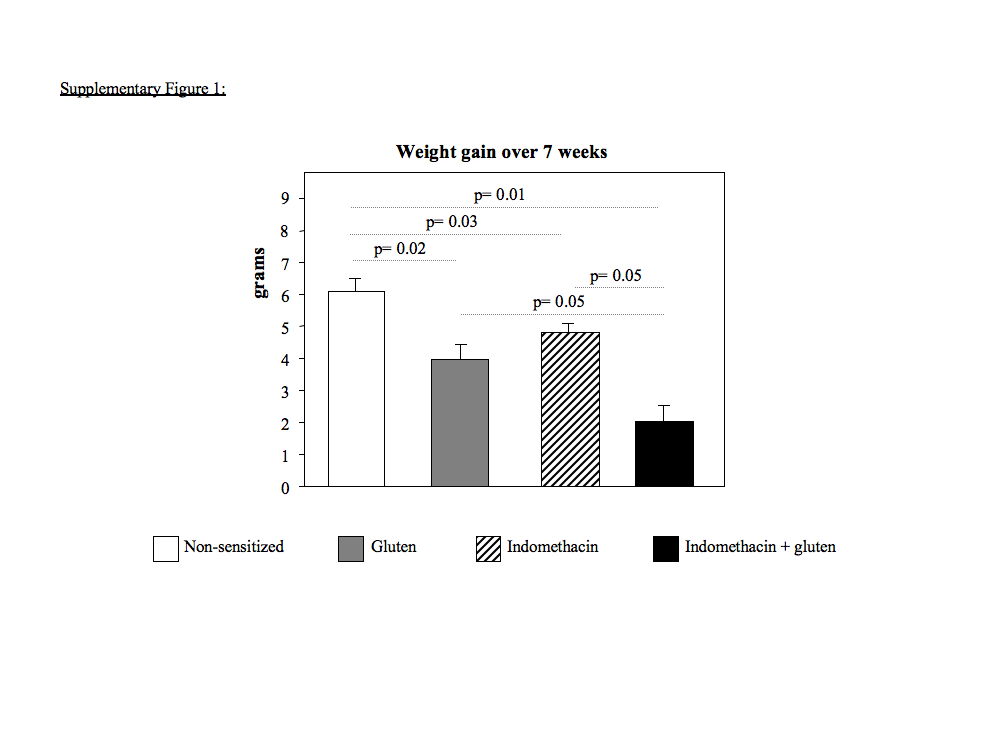

Supplement: Figure S1 — Weight gain over 7-week period. Both gluten-sensitized and indomethacin treated mice exhibited a decreased rate of weight gain compared to non-sensitized controls. Gluten-sensitized mice treated with indomethacin exhibited more pronounced weight gain retardation compared to controls and to gluten-sensitized and indomethacin treated mice. Data represent the means±SEM of 10 mice/group. (0.08 MB TIF) [file pone.0006472.s001.tif]

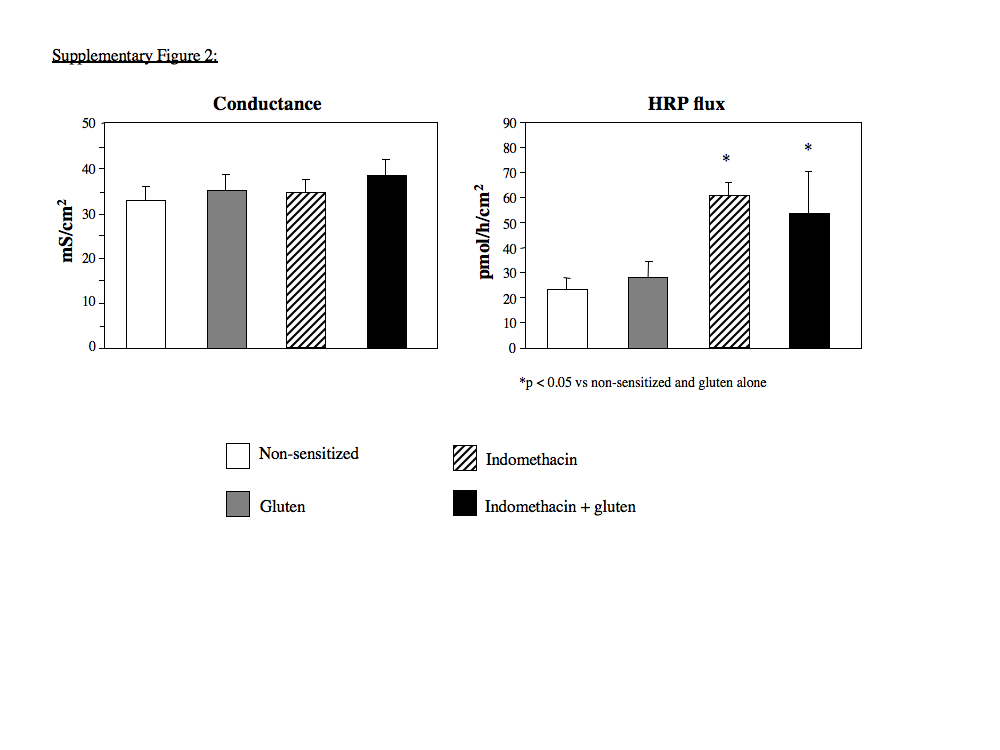

Supplement: Figure S2 — Conductance and HRP flux in C57Bl/6 mice. Gluten and/or indomethacin treatment did not lead to changes in tissue conductance. HRP flux was increased in indomethacin treated mice, but not in gluten sensitized mice without indomethacin. Data represent the means±SEM of 10 mice/group. (0.09 MB TIF) [file pone.0006472.s002.tif]

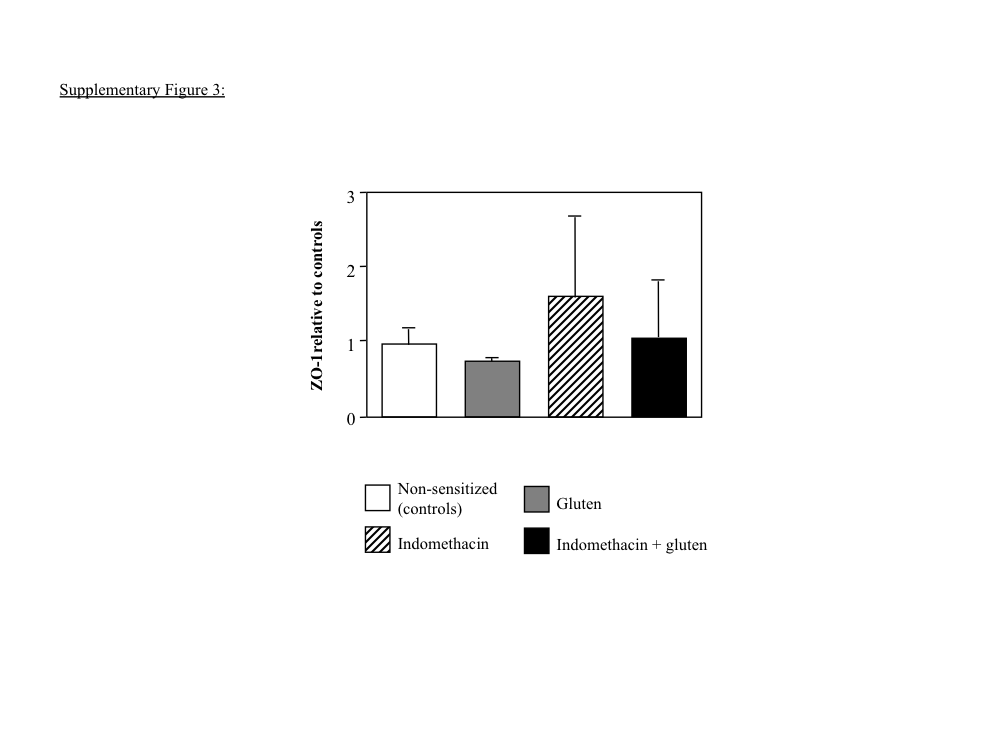

Supplement: Figure S3 — ZO-1 RNA expression relative to non-sensitized controls. No significant differences were seen when RNA expression for each treatment group was analyzed relative to non-sensitized controls. Data represent the means±SEM of 6 mice/group. (3.00 MB TIF) [file pone.0006472.s003.tif]

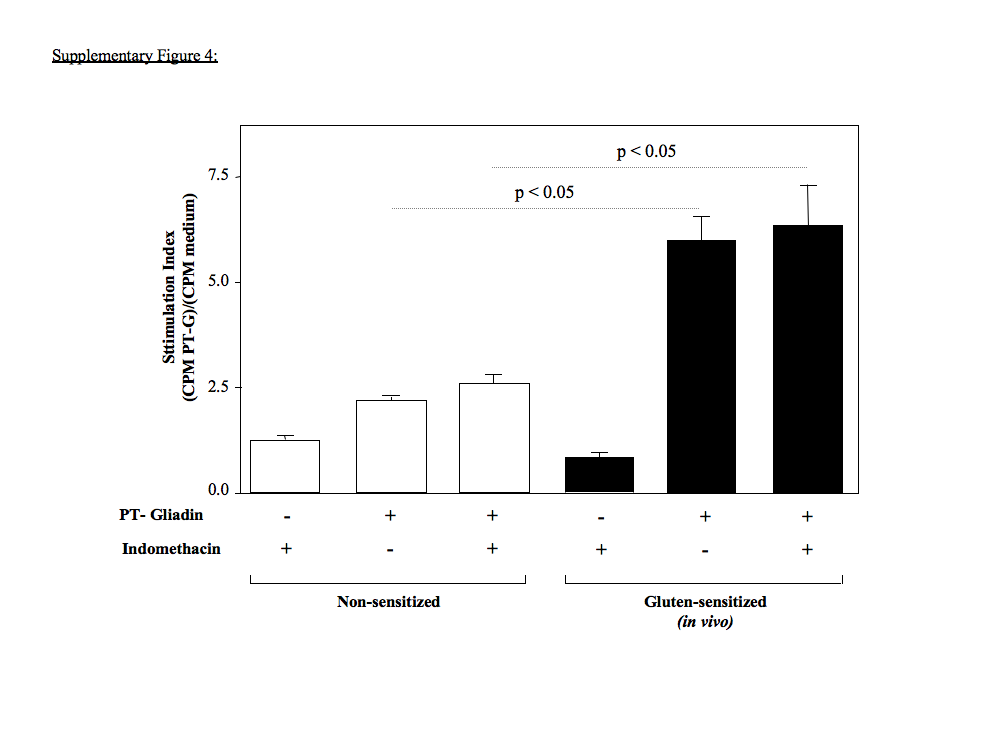

Supplement: Figure S4 — Splenocyte proliferation after incubation with PT-gliadin and/or indomethacin. Stimulation with indomethacin alone did not increase splenocyte proliferation in gluten-sensitized mice. In-vitro stimulation with both PT-gliadin and indomethacin, did not further enhance cell proliferation compared to PT-gliadin alone. Data represent the means±SEM of 6 mice/group. (0.07 MB TIF) [file pone.0006472.s004.tif]

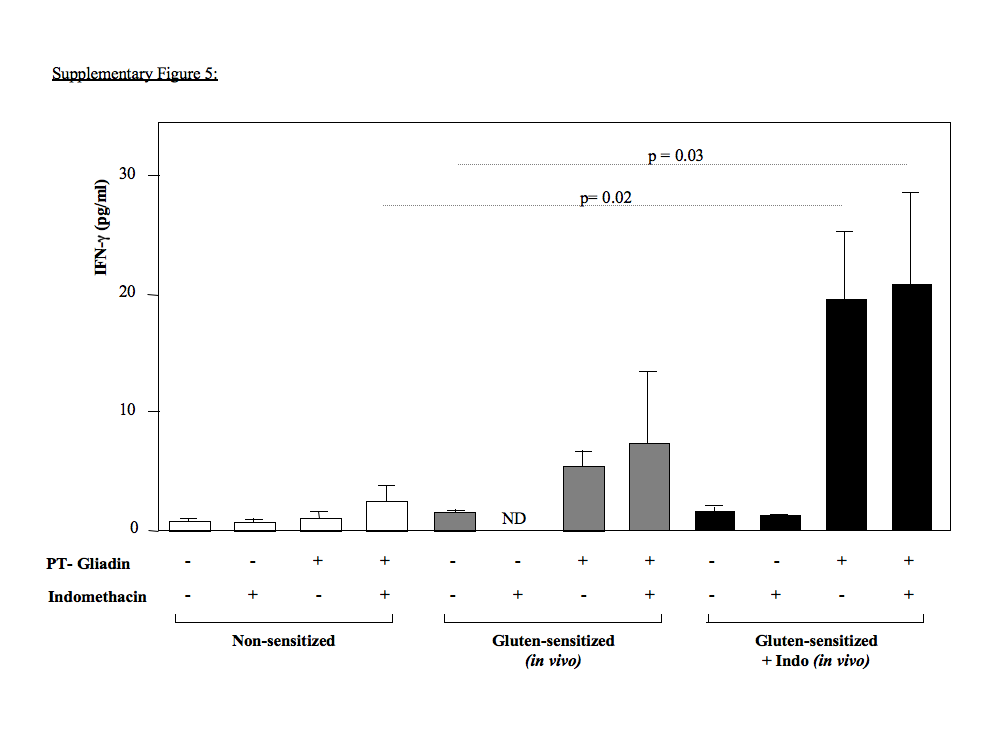

Supplement: Figure S5 — IFN-γ levels in supernatant of cultured splenocytes after incubation with PT-gliadin and/or indomethacin. Stimulation with indomethacin alone did not increase IFN-γ production in gluten-sensitized mice. In-vitro stimulation with PT-gliadin and indomethacin did not increase IFN-γ levels compared to PT gliadin alone. Data represent the means±SEM of 6 mice/group. ND = not detectable. (0.07 MB TIF) [file pone.0006472.s005.tif]

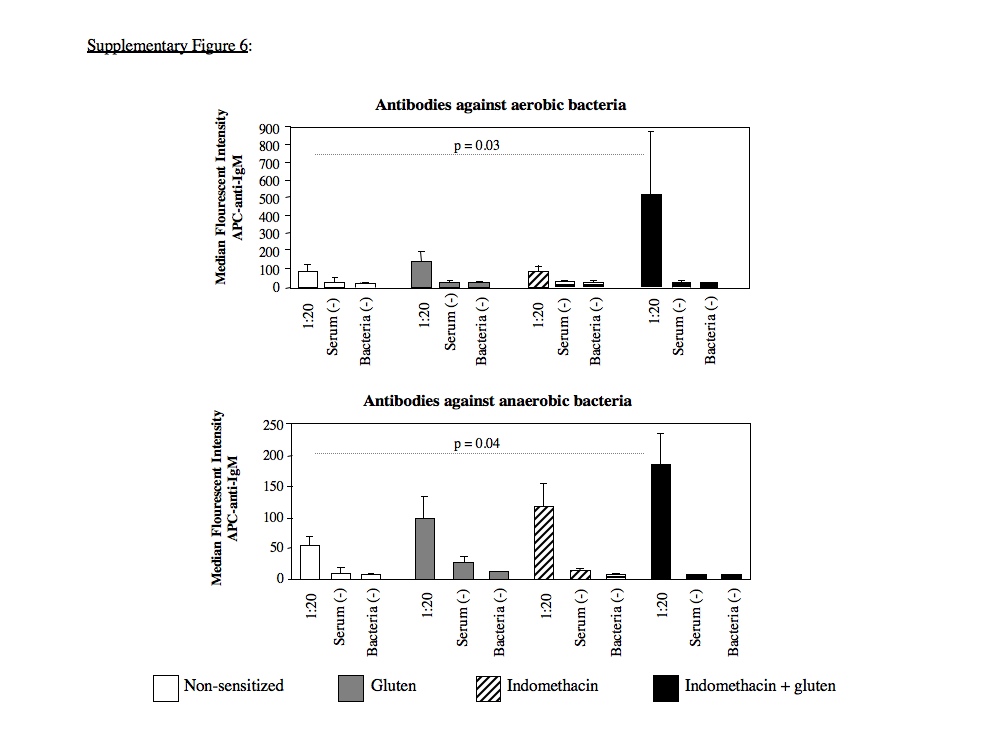

Supplement: Figure S6 — Systemic antibodies against commensals. Gluten-sensitized plus indomethacin-treated mice exhibited increased serum antibodies against aerobic and anaerobic bacteria as assessed by median fluorescent intensity signal of APC-labelled anti-IgM (1∶20 serum dilution). Negative controls include serum (−): no serum and bacteria (−): no bacteria. Data represent the means±SEM of 6 mice/group. (0.11 MB TIF) [file pone.0006472.s006.tif]

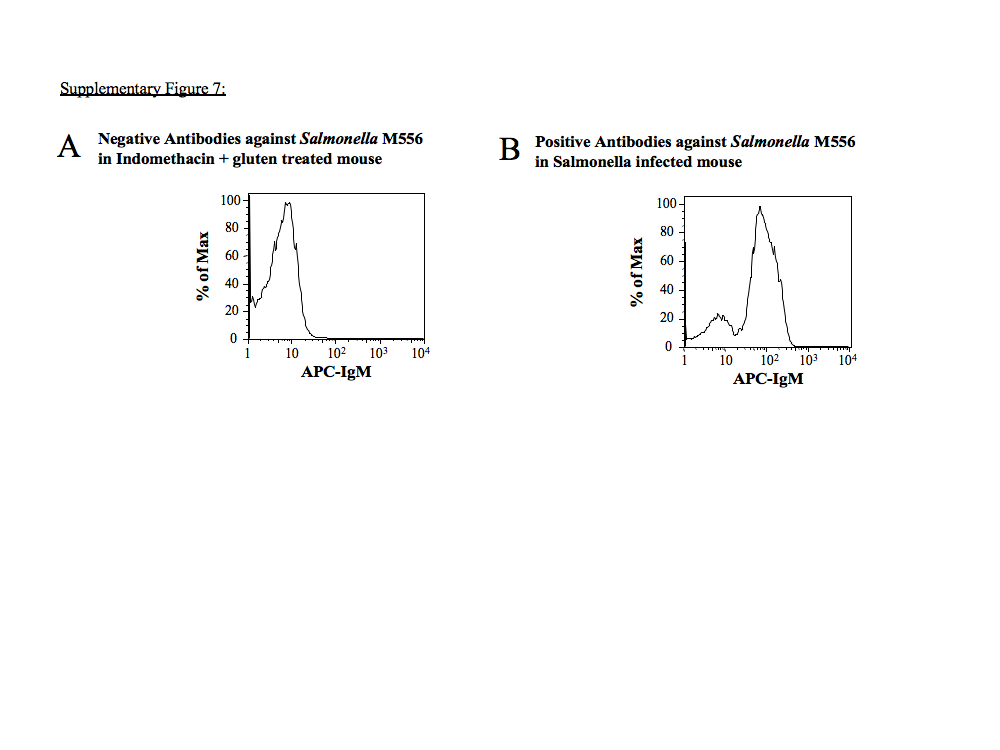

Supplement: Figure S7 — Positive and negative systemic antibodies against commensals. (A) Salmonella M557, which is a pathogen not present in our HLA-DQ8/HCD4 mice colony, was stained with serum antibodies from indomethacin plus gluten treated mice. Results show the absence of positive antibodies against Salmonella, thus the specificity of the technique. (B) Salmonella M557 was stained with serum antibodies from Salmonella M557 infected mice. Results show the absence of positive antibodies against Salmonella. (0.09 MB TIF) [file pone.0006472.s007.tif]

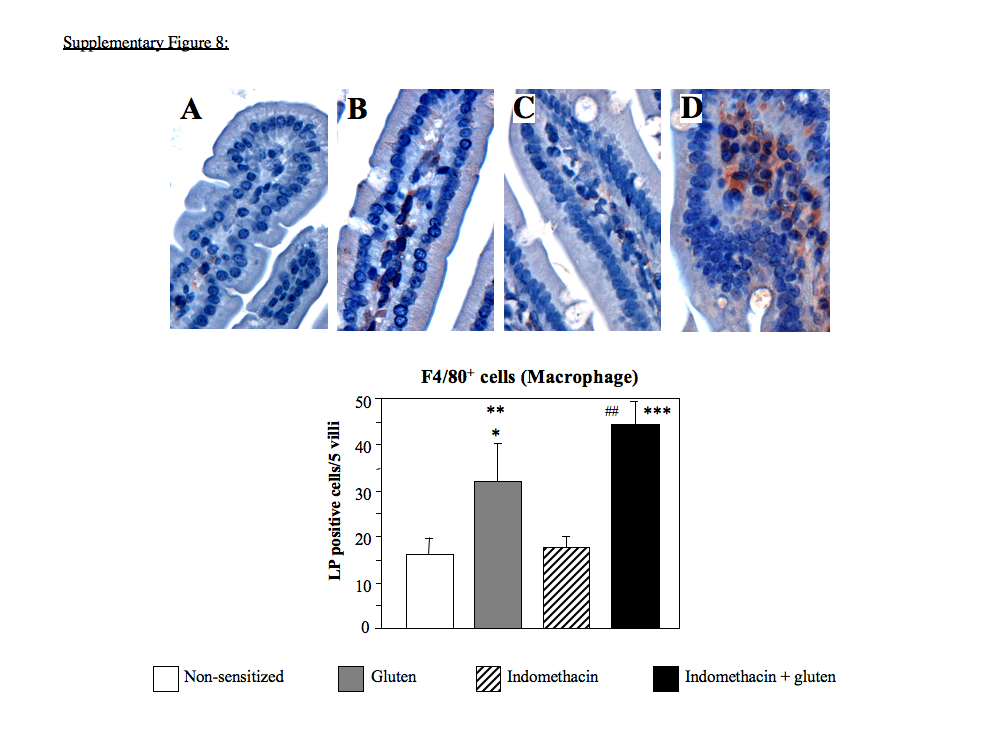

Supplement: Figure S8 — Immunohistochemistry for F4/80+ cells. Staining for F4/80+ was increased in gluten sensitized mice. Infiltration of F4/80+ cells was most marked in gluten-sensitized mice treated with indomethacin. Data represent the means±SEM of 6 mice/group. Representative picture of macrophage infiltration in the lamina propria from (A) control mice (B) gluten-sensitized mice (C) indomethacin-treated mice (D) gluten-sensitized plus indomethacin treated mice. (0.47 MB TIF) [file pone.0006472.s008.tif]
