# Supplementary material for: Host Responses to Intestinal Microbial Antigens in Gluten-Sensitive Mice
Source: PLoS One. 2009 Jul 31;4(7):e6472. doi: 10.1371/journal.pone.0006472 (PMC2715133; doi:10.1371/journal.pone.0006472)
Supplement: Table S1 — Oligonucleotide probes and hybridization conditions used in FCM-FISH analysis of intestinal bacteria. (0.06 MB DOC) [file pone.0006472.s009.doc]

**Supplementary Table 1.**

**Oligonucleotide probes and hybridization conditions used in FCM-FISH analysis of intestinal bacteria.**

| **Probe** | **Target bacterial group** | **Sequence (5’-3’)** | **References** |
| --- | --- | --- | --- |
| EUB338 | Domain bacteria | GCTGCCTCCCGTAGGAGT | [1] |
| NON338 | Negative control | ACATCCTAC GGGAGG C | [2] |
| Bif164 | *Bifidobacterium* | CATCCGGCATTACCACCC | [3] |
| Lab158 | *Lactobacillus-other lactic acid bacteria* | GGTATTAGCA(C/T)C TGT TTC CA | [4] |
| Bac303 | *Bacteroides-Prevotella* | CCAATGTGGGGGACCTT | [5] |
| Eco11513 | *Escherichia coli* | CACCGTAGTGCCTCGTCATCA | [6] |
| Elgc01 | *Clostridium leptum* | GGGACGTTGTTTCTGAGT | [7] |
| Erec0482 | *Eubacterium rectale/Clostridium coccoides* | GCTTCTTAGTCAGGTACCG | [8] |
| CHis150 | *Clostridium histolyticum* | TTATGCGGTATTAATCT(C/T)CCTTT | [9] |
| CLis135 | *Clostridium lituseburense* | GTTATCCGTGTGTACAGGG | [10] |
| SBR | *Sulphate-reducing bacteria* | TACGGATTTCACTCCT | [11] |

**References:**

[1] Nadal I, Donat E, Ribes-Koninckx C, Calabuig M, Sanz Y (2007) Imbalance in the composition of the duodenal microbiota of children with coeliac disease. J Med Microbiol 56:1669-1674.

[2] Sanz Y, Sanchez E, Marzotto M, Calabuig M, Torriani S et al (2007) Differences in faecal bacterial communities in coeliac and healthy children as detected by PCR and denaturing gradient gel electrophoresis. FEMS Immunol Med Microbiol 51:562-568.

[3] Langendijk PS, Schut F, Jansen GJ, et al. Quantitative fluorescence in situ hybridization of Bifidobacterium spp. with genus-specific 16S rRNA-targeted probes and its application in fecal samples. Applied and Environmental Microbiology 1995 Aug;61(8):3069-3075.

[4] Harmsen HJ, Wildeboer-Veloo AC, Grijpstra J, et al. Development of 16S rRNA-based probes for the Coriobacterium group and the Atopobium cluster and their application for enumeration of Coriobacteriaceae in human feces from volunteers of different age groups. Applied and Environmental Microbiology 2000 Oct;66(10):4523-4527.

[5] Manz W, Amann R, Ludwig W, et al. Application of a suite of 16S rRNA-specific oligonucleotide probes designed to investigate bacteria of the phylum cytophaga-flavobacter-bacteroides in the natural environment. Microbiology (Reading, England) 1996 May;142 ( Pt 5)(Pt 5):1097-1106.

[6] Poulsen LK, Lan F, Kristensen CS, et al. Spatial distribution of Escherichia coli in the mouse large intestine inferred from rRNA in situ hybridization. Infection and immunity 1994 Nov;62(11):5191-5194.

[7] Franks AH, Harmsen HJ, Raangs GC, et al. Variations of bacterial populations in human feces measured by fluorescent in situ hybridization with group-specific 16S rRNA-targeted oligonucleotide probes. Applied and Environmental Microbiology 1998 Sep;64(9):3336-3345.

[8] Hold GL, Schwiertz A, Aminov RI, et al. Oligonucleotide probes that detect quantitatively significant groups of butyrate-producing bacteria in human feces. Applied and Environmental Microbiology 2003 Jul;69(7):4320-4324.

[9] Bullock NR, Booth JC, Gibson GR. Comparative composition of bacteria in the human intestinal microflora during remission and active ulcerative colitis. Current Issues in Intestinal Microbiology 2004 Sep;5(2):59-64.
