# Supplementary material for: Host Responses to Intestinal Microbial Antigens in Gluten-Sensitive Mice
Source: PLoS One. 2009 Jul 31;4(7):e6472. doi: 10.1371/journal.pone.0006472 (PMC2715133; doi:10.1371/journal.pone.0006472)
Supplement: Protocol S1 — Immunohistochemistry for macrophages. (0.03 MB DOC) [file pone.0006472.s010.doc]

**Protocol S1. Immunohistochemistry for macrophages**

Immunostaining for macrophages was performed on paraffin sections using a monoclonal antibody recognizing the F4/80 antigen [1]. The primary antibody rat anti-mouse (1:2000; Serotec, London) was followed by biotinylated polyclonal goat anti-rat antibody (1:200; Cederlane Laboratories, Ontario) and then streptavivdin/HRP (1:300; Dakocytomation, Ontario). Antibodies were visualized by DAB and counterstaining with Mayer’s hematoxylin. Negative controls were performed in the absence of primary antibody.

**Reference:**

[1] Bercík P, De Giorgio R, Blennerhassett P, Verdú EF, Barbara G, Collins SM. Immune-mediated neural dysfunction in a murine model of chronic Helicobacter pylori infection. Gastroenterology. 2002 Oct;123(4):1205-15.
